# The specific signs of lung ultrasound to diagnose pulmonary hemorrhage of the newborns: Evidence from a multicenter retrospective case-control study

**DOI:** 10.3389/fped.2023.1090332

**Published:** 2023-01-18

**Authors:** Jing Liu, Jing-Han Chi, Zu-Lin Lu, Wei Fu

**Affiliations:** ^1^ Department of Neonatology and NICU, Beijing Chao-Yang Hospital, Capital Medical University, Beijing, China; ^2^ Department of Pediatric Medicine, the Seventh Medical Center of PLA General Hospital, Beijing, China; ^3^ Department of Neonatology and NICU, Beijing Chaoyang District Maternal and Child Healthcare Hospital, Beijing, China

**Keywords:** lung ultrasound, pulmonary hemorrhage, neonate, specific ultrasound sign, fluid bronchogram, fibrin deposition sign

## Abstract

**Objectives:**

Pulmonary hemorrhage (PH) is one kind of critical lung diseases in newborn infants, which is the most difficult one to be diagnosed by ultrasound. This study was to investigate the specific ultrasonic signs of PH in order to better diagnose neonatal PH by using lung ultrasound (LUS).

**Methods:**

A total 168 newborn infants were enrolled in this study, which included PH, pneumonia, meconium aspiration syndrome, and newborns without lung diseases, there were 42 cases in each group. In a quiet state, infants were placed in the supine, lateral or prone position for the examination. Each lung was divided into the anterior, lateral and posterior regions, then each region of both lungs was scanned with the probe perpendicular to the ribs or parallel to the Intercostal spaces.

**Results:**

The major results showed that: (1) the common LUS manifestation of PH includes lung consolidation, air bronchograms, fluid bronchograms, pleural effusion, shred signs, pleural line abnormality and B-lines, while fibrin deposition sign is a rare sign of PH. (2) Co-existing of lung consolidation with fluid bronchograms and pleural effusion is the specific sign of PH with a sensitivity of 81.0%, specificity of 98.4% and the positive predictive value (PPV) was 94.4%. (3) Fibrin deposition sign is an uncommon specific LUS sign of PH with a sensitivity 28.6%, specificity of 100% and the PPV was 100%. (4) Nine patients (21.4%) were diagnosed with PH based on ultrasound findings before oronasal bleeding. (5) The survival rate of infants with PH was 100% in this study.

**Conclusion:**

LUS is helpful for the early diagnosis of neonatal PH and may therefore improve the prognosis. The lung consolidation with fluid bronchograms and pleural effusion as well as fibrin deposition sign are specific to diagnose PH by using LUS.

## 1. Introduction

Pulmonary hemorrhage (PH) is one kind of severe pathological disorders that associated with higher morbidity and mortality in newborn infants, particularly among preterm infants stay in neonatal intensive care unit (NICU) (1, 2). It was reported that the prevalence of neonatal PH was 6.7 for 1,000 live births, the rates observed were 8% among newborns <1,500 g, and 11% among newborns <1,000 g (3). Once PH occurs, the fatality rate can be increased as high as 56.25% (4), while the survivors also had a higher incidence of bronchopulmonary dysplasia (BPD), cerebral palsy and cognitive delay increased 2.5 times in premature infants with PH, and the incidence of severe periventricular leukomalacia also increased significantly (5, 6). Early diagnosis and early treatment are the key to improve the prognosis of PH infants. Traditionally, the diagnosis is made when hemorrhagic secretions are aspirated from the trachea concurrent with respiratory decompensation that necessitates intubation or escalated support (7). However, diagnosis at this stage is often too late to improve the prognosis of patients. Chest x-ray (CXR) or even computed tomography (CT) has been used for PH diagnosis, however, it still has no specificity and can not be diagnosed earlier (7). In addition, CXR or CT causes inevitable damage to the human body, especially to newborn infants who are in the growing and developing is more serious. A much recent long-term follow-up study showed that for every 10 mGy increase in the dose of radiation received in childhood, the risk of central nervous system tumors increased 1.05 times (95% CI: 1.01–1.09), increased the risk of leukemia by 1.17 times (95% CI: 1.09–1.26) (8). Therefore, it is of great significance to explore the early diagnostic techniques without radiation damage for PH in newborn infants.

Recently, as a kind of harmless “green” diagnostic technique, lung ultrasound (LUS) has been widely used in the diagnosis and differential diagnosis of neonatal lung diseases (9–12). In addition, we found that LUS can diagnose PH before blood emerging from the mouth and nose among some patients, that is, LUS maybe has early diagnostic value for PH (12). With the further study of LUS, it was found that certain ultrasound signs are highly specific for the diagnosis of neonatal PH. In this paper, we will focus on these specific ultrasound signs used for diagnosing PH, so that we can make an early diagnosis of neonatal PH by using LUS.

## 2. Patients and methods

### 2.1. Patients

Forty-two consecutive infants with PH from May 2017 to December 2021 were included in this study. According to our clinical practice and literatures, the PH, pneumonia and meconium aspiration syndrome (MAS) can have some similar ultrasound manifestations, such as lung consolidation with air bronchograms (12–17). Therefore, to determine whether some of the ultrasound findings are specific to PH or not, the controls will include three subgroups, that were pneumonia group, MAS group and normal lungs, respectively. To minimize statistical error, the sample size in each subgroup was randomly selected at a ratio of 1:1. Thus, a total of 168 patient's (that were PH 42 cases, pneumonia 42 cases, MAS 42 cases and 42 normal lung controls) were included in this case-control study.

The Diagnostic criteria for neonatal PH were as following (3–7): (1) Infants often present with dyspnea, cyanosis and hypoxia exacerbation suddenly that necessitates intubation or escalated support. (2) The fine moist rales in the lungs suddenly significantly increased on auscultation. (3) The most important evidence for PH diagnosis is the hemorrhagic secretions from the infant's mouth, nose, or endotracheal tube.

The exclude criteria for neonatal PH was that the bleeding was caused by airway injuries when tracheal aspiration. Generally, we can distinguish them by the following aspects: (1) The amount of bleeding during tracheal injury is generally less. (2) Infant's dyspnea is usually not substantially deterioration. (3) There is also usually no significant change in pulmonary signs on auscultation.

## 3. Methods

### 3.1. Ultrasound instruments and examination methods

A high-frequency (10–14 MHz) linear probe (Voluson S10, GE Medical Systems, Kretz, Austria) was used for ultrasound examinations (15, 18). LUS was routinely performed in every infant with dyspnea on admission or exacerbation of dyspnea during hospitalization. The patient may be in the supine, prone, or lateral position during LUS examination. There is no need to interrupt the ventilator treatment when examination. An assistant should be available when changing the body position of the infant during the examination. According to the guidelines (15, 18), each lung was divided into the anterior, lateral and posterior regions by the anterior axillary and posterior axillary lines. Each region of both lungs was scanned carefully with the probe perpendicular to the ribs or parallel to the Intercostal spaces. All of the ultrasound findings were recorded carefully.

### 3.2. Statistical analysis

The Statistical Package for the Social Sciences (SPSS) 24.0 software was used to statistically analyze the data. The Chi-squared test was used to compare the positive rates of the ultrasound findings in each group, and the specificity and sensitivity of the major ultrasound signs for the diagnosis of neonatal PH were calculated. A value of *p* < 0.05 indicated statistically significant differences.

## 4. Results

### 4.1. The demographic data in the four groups

The general demographic data of the four groups are shown in Table 1.

**Table 1:** General demographic data in different groups.

Groups	*n*	Male/Female	GA (weeks)	Birth weight (g)	Premature/Term	VD/CD
PH	42	22/20	28^+2^–40^+6^	980–4,650	19/23	25/17
MAS	42	23/19	32^+3^–41^+3^	1,550–4,080	5/37	27/15
Pneumonia	42	21/21	28^+5^–39^+4^	950–3,990	24/18	27/15
Normal control	42	19/23	29–39^+1^	1,200–4,440	22/20	26/16

PH, pulmonary hemorrhage; GA, gestational age; VD, viginal delivery; CD, Cesarean delivery.

### 4.2. The onset time of neonatal PH

The onset time of all the PH was within 1 week after birth. 34 cases (81%) of them occurred with in 2 h of life; In 6 h of life, the case of PH increased to 36 (85.7%); In 24 h of life, the case of PH increased to 38 cases (90.5%); In 72 h of life, more than the case of PH increased to 41 (97.6%); After 72 h of birth, only one infant left occurred PH (2.4%). The earliest patient onset occurs within 10 min of birth.

### 4.3. LUS manifestations of neonatal PH

**Figure 1:**
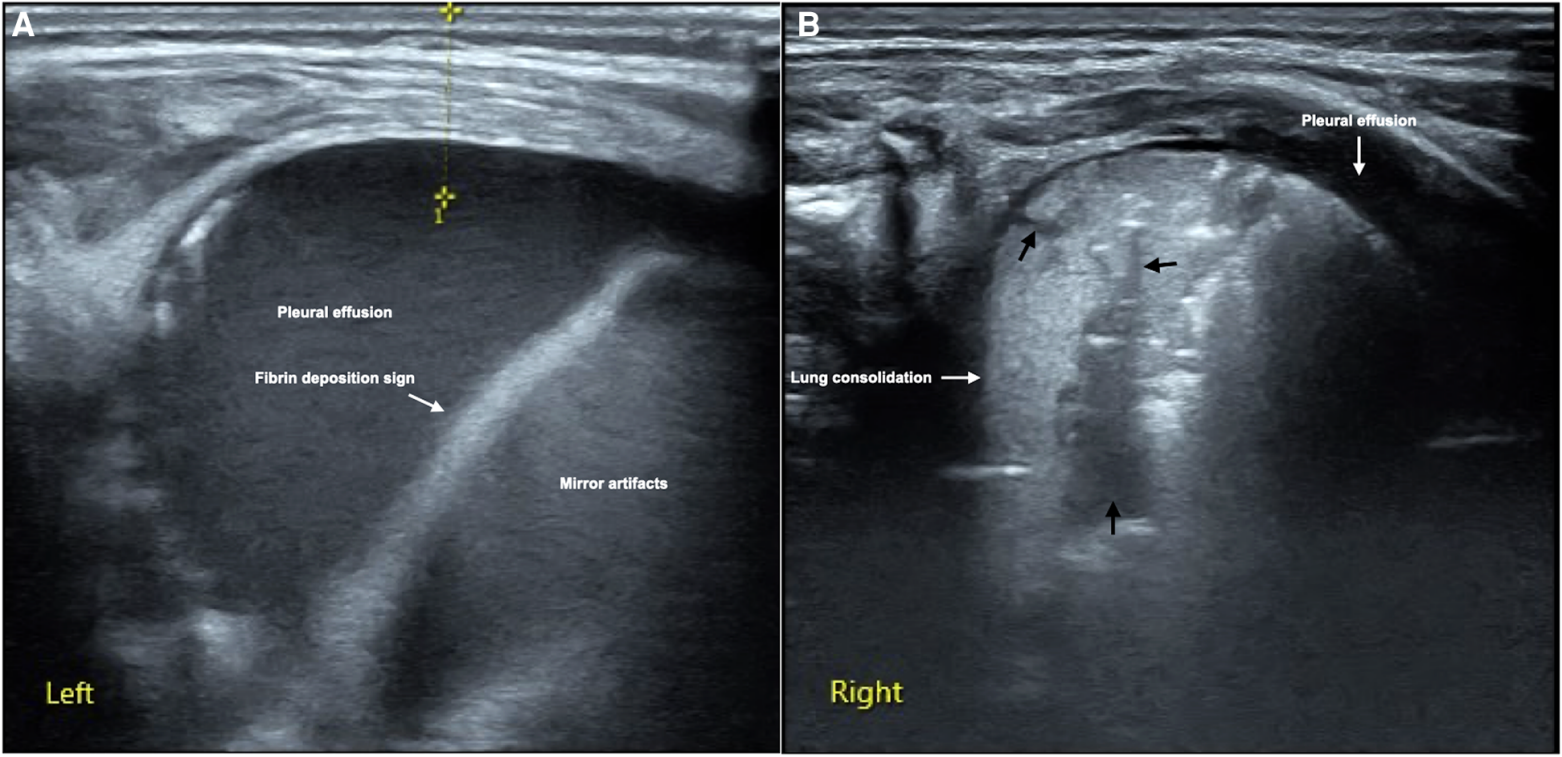
LUS manifestations of neonatal PH. This is a male term infant delivered by cesarean section at gestational age 40^+3^ weeks with birth weight of 3,890 g. He was admitted to NICU at 10 min of birth due to fetal distress, severe birth asphyxia and dyspnea after resuscitation. Physical examination found that the infant's respiratory rate increased by more than 70/min with significant retraction, palpable wet rales on auscultation. LUS showed massive pleural effusion and mirror artifacts, and fibrin deposition sign in the left thoracic cavity (**A**), while the right lung should significant lung consolidation accompanied with fluid bronchograms (thick black arrows), and a small amount of pleural effusion (**B**). 30 ml of hemorrhagic effusion was extracted from the left thoracic cavity by pleural puncture.

**Figure 2:**
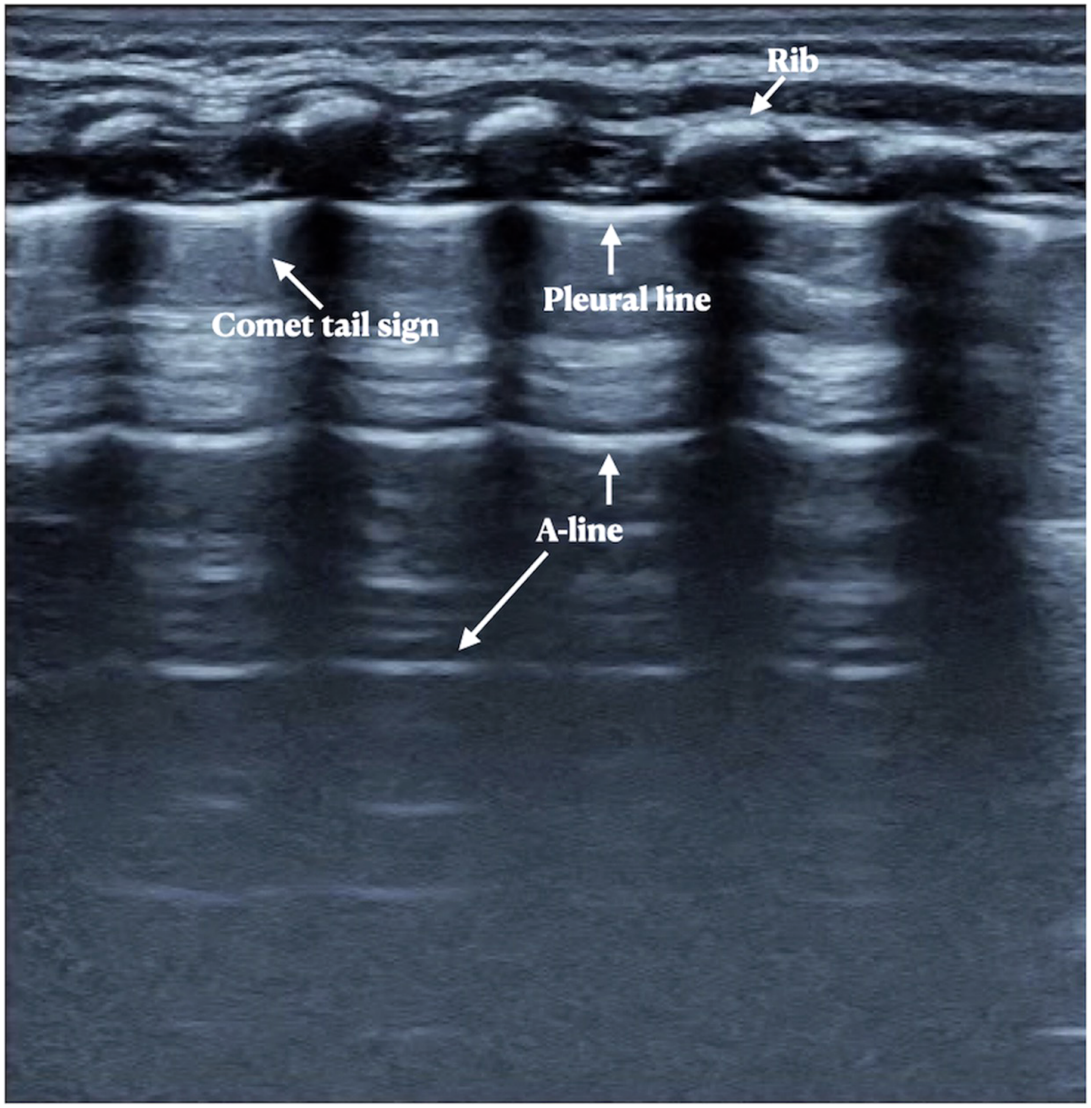
Neonatal normal lung ultrasound findings. On B-mode imaging, the neonatal normal lung presents as the bamboo sign. The pleural line and A-line present as smooth, regular and hyperechoic lines arranged in parallel and equidistant from each other, and the A-line echoes gradually diminish until disappeared. In those infdants less than a week after birth, a few B-lines or comet tail signs may sometimes be seen, but there is no pleural effusion or lung consolidation.

It can be see from Table 2, the major and common LUS manifestations are lung consolidation (92.9%), air bronchograms (92.9%), fluid bronchograms (81%), pleural effusion (85.7%), shred signs (83.3%), B-lines (100%) and pleural line abnormalities (100%). The fibrin deposition sign is a rare ultrasonic sign of PH, which was found among 12 patients (28.6%) (Figure 1). Sometimes, dynamic fluid bronchograms (DFB) could be observed in severe PH patients under real-time ultrasound (Supplementary Video S1). While in normal lungs, only a few infants can have a small number of B-lines, but with no lung consolidation and pleural effusion (Figure 2).

**Table 2:** The LUS performance of PH compared with the control group (*n*, %).

Ultrasound signs	PH (42, %)	MAS (42, %)	Pneumonia (42, %)	Normal lung (42, %)
Lung consolidation	39 (92.9)	42 (100)	42 (100)	0 (0)
Air bronchograms	39 (92.9)	42 (100)	40 (95.2)	0 (0)
Fluid bronchograms	34 (81.0)	1 (2.38)	1 (2.38)	0 (0)
Pleural effusion	36 (85.7)	2 (4.76)	3 (7.14)	0 (0)
Fibrin deposition sign	12 (28.6)	0 (0)	0 (0)	0 (0)
Shred signs	35 (83.3)	30 (71.4)	31 (73.8)	0 (0)
B-lines	42 (100)	42 (100)	42 (100)	5 (11.9)
Pleural line abnormalities	42 (100)	42 (100)	42 (100)	0 (0)

PH, pulmonary hemorrhage.

### 4.4. The sensitivity and specificity of some ultrasound signs for PH diagnosis

#### 4.4.1. Co-existing of lung consolidation with fluid bronchograms and pleural effusion

As can be seen from Table 2, although lung consolidation, B-lines and pleural line are the common signs of PH, they are also the common findings in other kinds of lung diseases such as MAS and pneumonia, etc., so they have no specific value for the diagnosis of neonatal PH. Fluid bronchograms and pleural effusion are also seen in diseases such as MAS and pneumonia, but they most frequently been seen in PH. In addition, fluid bronchograms only appear in the consolidated lung tissues, so we used lung consolidation, fluid bronchograms and pleural effusion to calculate their specificity and sensitivity for the diagnosis of PH of newborns in this study. As can be seen from Table 3, when these threes signs are co-existed, the sensitivity and specificity for diagnosing PH were 81.0% and 98.4% with a positive predictive value (PPV) was 94.4%, respectively.

**Table 3:** The sensitivity and specificity of co-existing signs for PH diagnosis.

Co-existing sign^a^	PH	Controls^b^	Total	Sensitivity (%)	Specificity (%)	PPV (%)
Present	34	2	36	81.0	98.4.	94.4
Not present	8	124	132			
Total	42	126	168			

PH, pulmonary hemorrhage; PPV, positive predictive value.

^a^ Includes lung consolidation, fluid bronchograms and pleural effusion.

^b^ Includes MAS, pneumonia and normal controls.

#### 4.4.2. Fibrin deposition sign

It has been confirmed that pleural effusion in infants with PH was confirmed as bloody fluid by pleural puncture (12) (Figure 1). In the pleural effusion of a few patients with severe PH, the fiber strip-like shadow and its artifacts formed by the destruction of blood cells and fibrin deposition could be seen, which was called the fibrin deposition sign (10) (Figure 1). The fibrin deposition sign can be seen moving with the fluctuation of the fluid under real-time ultrasound (Supplementary Video S2). Although this sign is uncommon, it is only seen in PH patients while not in other kinds of lung diseases (such as MAS and pneumonia) and normal lungs in the present study, therefore, this sign has specific value for the diagnosis of PH. As we can seen from Table 4, although the sensitivity of this sign in the diagnosis of PH is only 28.6%, the specificity is as high as 100% with the PPV was also 100%.

**Table 4:** The sensitivity and specificity of fibrin deposition sign for PH diagnosis.

Fibrin deposition sign	PH	Controls^a^	Total	Sensitivity (%)	Specificity (%)	PPV (%)
Present	12	0	12	28.6	100	100
Not present	30	126	156			
Total	42	126	168			

PH, pulmonary hemorrhage; PPV, positive predictive value.

^a^ Includes MAS, pneumonia and normal controls.

### 4.5. Correlation between LUS findings and oronasal bleeding in PH patients

In this study, nine patients (21.4%) were suspected as PH due to the presence of the lung consolidation and significant fluid bronchograms, or lung consolidation with fluid bronchograms and pleural effusion before oronasal bleeding was found. After endotracheal intubation, the sputum suction tube is inserted into the deep trachea, and then the fresh bloody fluid is sucked out under negative pressure.

### 4.6. Outcomes of PH patients

No PH patient died in this study, that is to say, all the PH patients were cured and discharged. Therefor, the mortality of this group PH patient is 0%. In addition, no BPD, no periventricular leukomalacia, etc. occurred in all of the PH patients.

## 5. Discussion

Generally, the onset of neonatal PH is early. The results of this study showed that more than 80% of PH occurred with in 2 h, more than 85% occurred with in 6 h, more than 90% occurred with in 24 h and more than 97% occurred with in 72 h of life, the earliest onset occurs within 10 min of birth. Therefore, if an infant has dyspnea soon after birth, the possibility of pulmonary hemorrhage should be considered. The survival rate was 100% and there was no poor prognosis in this study after timely and accurate diagnosis and reasonable treatment. In this study, 21.4% of the PH patients were diagnosed by their LUS findings before oronasal bleeding occurs confirming that LUS is helpful for the early diagnosis of PH in newborn infants. So we believe that managing neonatal PH under LUS monitoring has some advantages: (1) LUS can make an early (that is before bloody fluid flows from the mouth and nose) diagnosis of PH. (2) Under LUS monitoring, ventilator parameters or ventilation mode can be adjusted timely according to the degree of lung lesions, so as to achieve the best ventilation effects. (3) It can timely find pleural effusion and timely performing puncture drainage, so as to promote the recovery of PH.

According to the results of this study, the common LUS manifestation of PH includes lung consolidation, air bronchograms, fluid bronchograms or even dynamic fluid bronchograms in severe PH patients under real-time ultrasound (Supplementary Video S1), pleural effusion, shred signs, pleural line abnormalities and different degree of lung edema, while the fibrin deposition sign is the rare ultrasonic sign of PH. The causes of lung consolidation during PH may be as the followings: (1) Alveolar occlusion and atelectasis resulting from blood blocking the alveoli and terminal bronchioles. (2) Lung consolidation associated with the primary disease that causing neonatal PH. According to the results of this study the fluid bronchograms are most commonly seen in PH, although they can be seen in other lung diseases such as pneumonia or MAS (Table 2), which may be caused by the rupture of alveolar capillaries and bleeding or inflammatory secretions into the peripheral bronchioles. Most of the PH infants have pleural effusion and it is confirmed to be bloody fluid by pleural puncture, which has not been recognized before LUS was used clinically. Therefore, the development of LUS technology changes our traditional recognition of PH, and is helpful to improve its prognosis. We believe that the possible reasons for pleural effusion in patients with PH are: (1) Intrapulmonary hemorrhage enters the pleural space through the terminal bronchioles. From the PH images, we can find the fluid bronchogram signs within the lung consolidation areas extending to the pleural cavity. (2) The permeability of the pleura increases when PH occurs, and intrapulmonary hemorrhage exuded into the pleural space. According to our present study and long-term clinical experience, a large pleural effusion in PH requires pleural puncture under ultrasound monitoring to drain the effusion, which can reduce patient's dyspnea and facilitate their recovery. Otherwise, the high mortality from PH is almost inevitable (3–6).

Among all the neonatal lung diseases, PH is the most difficult one to be diagnosed by ultrasound. According to the results of this study, some specific ultrasound signs are helpful to the ultrasonic diagnosis of PH. These specific ultrasound signs include: (1) Fibrin deposition sign: although its sensitivity in diagnosis of PH is only 28.6%, the specificity is as high as 100% (Table 4). (2) Co-existing of lung consolidation with fluid bronchograms and pleural effusion: the results of this study showed when these the signs are presented together, the sensitivity and specificity for diagnosing PH are 81.0% and 98.4%, respectively. Besides, fluid bronchograms are occasionally seen in other lung diseases such as MAS or pneumonia, but are most common in PH. More than 80% of PH infants have this sign. Therefore, when fluid bronchograms are found when LUS examination, the possibility of PH should be considered firstly. If there is pleural effusion co-existed, the diagnosis of PH can be basically confirmed. Understanding and mastering these ultrasound signs is of great value in diagnosing PH using LUS.

In conclusion, the present study involved a more in-depth investigation of the ultrasound characteristics of neonatal PH and clarified the various ultrasound manifestations of PH, in particular, combined with typical imaging or videos, the specific signs of PH are introduced to help clinical or ultrasound professionals better diagnose PH with LUS. LUS is not only helpful for early diagnosis of neonatal PH and may therefore improve the prognosis of the patients, which can also avoid radiation hazards for PH patients, other infants in the same ward and medical staff. Therefor, it is worthy of routine application in neonatal field (19–21).

This study may also had some limitations due to the inclusion of a non-consecutive sample that may not be fully representative of all PH patients. Any one who would like to perform LUS should be properly trained, correctly understand the common used terminologies and concepts of LUS, master the operation methods of LUS and basic adjustment skills of instruments and get some experience (13, 18, 22). As a follow-up, we aim to design a prospective study that compares neonatal LUS to the final diagnosis reached by a blinded panel.

## Data Availability

The original contributions presented in the study are included in the article/**Supplementary Material**, further inquiries can be directed to the corresponding author.
